# Increased Detection of Sin Nombre Hantavirus RNA in Antibody-Positive Deer Mice from Montana, USA: Evidence of Male Bias in RNA Viremia

**DOI:** 10.3390/v5092320

**Published:** 2013-09-24

**Authors:** Karoun H. Bagamian, Jonathan S. Towner, James N. Mills, Amy J. Kuenzi

**Affiliations:** 1 Viral Special Pathogens Branch, National Center for Emerging and Zoonotic Infectious Diseases, Centers for Disease Control and Prevention, Atlanta, GA 30033, USA; 2 Department of Biology, Montana Tech, University of Montana, Butte, MT 59701, USA; E-Mails: jit8@cdc.gov (J.S.T.); akuenzi@mtech.edu (A.J.K.); 3 Population Biology, Ecology & Evolution Program, Emory University, Atlanta, GA 30322, USA; E-Mail: wildlifedisease@gmail.com

**Keywords:** Sin Nombre hantavirus, deer mouse, *Peromyscus maniculatus*, transmission, viral RNA, RT-PCR

## Abstract

Hantaviruses are widespread emergent zoonotic agents that cause unapparent or limited disease in their rodent hosts, yet cause acute, often fatal pulmonary or renal infections in humans. Previous laboratory experiments with rodent reservoir hosts indicate that hantaviruses can be cleared from host blood early in the infection cycle, while sequestered long term in various host organs. Field studies of North American deer mice (*Peromyscus maniculatus*), the natural reservoir of Sin Nombre hantavirus, have shown that viral RNA can be transiently detected well past the early acute infection stage, but only in the minority of infected mice. Here, using a non-degenerate RT-PCR assay optimized for SNV strains known to circulate in Montana, USA, we show that viral RNA can be repeatedly detected on a monthly basis in up to 75% of antibody positive deer mice for periods up to 3–6 months. More importantly, our data show that antibody positive male deer mice are more than twice as likely to have detectable SNV RNA in their blood as antibody positive females, suggesting that SNV-infected male deer mice are more likely to shed virus and for longer periods of time.

## 1. Introduction

Hantaviruses (family *Bunyaviridae*, genus *Hantavirus*) are negative-stranded RNA viruses that occur across Europe, Asia, and the Americas and are hosted by rodents, insectivores, and possibly bats [1,2]. The earliest studies of experimentally infected Old World hantavirus rodent reservoir hosts suggest that after inoculation, the animals experience a brief viremia 7–10 days post-infection (PI), before developing neutralizing immunoglobulin G (IgG) antibody approximately 10–21 days PI and clearing infectious virus from the blood [3,4]. Virus was sequestered in various tissues including lungs, liver, spleen, kidneys, and adipose tissue, resulting in persistent infection [3,4]. Laboratory studies of New World Sin Nombre virus (SNV) in its host, the deer mouse (*Peromyscus maniculatus*) demonstrated SNV viral antigen in tissues and viral RNA in blood for up to 217 days post-inoculation [5,6]. Previous field studies of SNV-infected deer mouse populations in Montana, USA and Canada have shown that 19%–45% of infected mice have intermittently detectable RNA in blood past the initial 10 day acute phase, sometimes up to three months [7,8]. In those studies, the authors suggested that either SNV-infected deer mice experience periodic episodes of virus recrudescence or that the level of viral RNA in the blood was consistently present at low levels, but hovered near the threshold of detection and often dropped below it. Support for the later scenario came from a recent, SNV field transmission study among *P. maniculatus* in outdoor enclosures, in which viral RNA was consistently detected in weekly blood samples up to 60 days PI using a newly developed and highly sensitive real-time RT-PCR assay that targeted a conserved region of the genome [9]. In addition, donor mice that successfully infected susceptible mice tended to have higher levels of viral RNA circulating in their blood [9], suggesting that presence of viral RNA may be a useful indicator of infectiousness. 

In many naturally occurring hantavirus rodent reservoir host systems, adult males are more likely to be antibody positive (Ab+) than females [10,11,12], and for SNV in *P. maniculatus*, males are three times more likely to seroconvert from antibody negative (Ab-) to Ab+ than females [13]. This observation has thus far been explained by the discovery of a relationship between indicators of aggression (such as presence, frequency, and/or severity of wounding) and the presence of hantavirus antibody in male hosts [9,14,15,16]. As a result, male deer mice are thought to be the main drivers of SNV transmission in natural systems, based primarily on differences in behavior. Specifically, as male deer mice defend their territories and compete with other males [17], they are more likely to fight and transmit virus, presumably through infectious saliva [18]. While differences in virus shedding between males and females have been reported for the Old World Seoul hantavirus (SEOV)-Norway rat (*Rattus norvegicus*) system, experimental infection studies of SNV in deer mice have thus far not reported similar findings after examining tissue, saliva, and excreta of infected mice [6,18,19]. Further, there have been only a few reports categorizing by sex the presence of SNV RNA in blood samples from naturally infected mice [7,8]. As a result, it is unclear if infected male mice are more likely than females to transmit virus due to differences in behavior or if there is an innate, sex-based SNV infection dynamic that allows infected males to remain ‘viremic’ (RNA positive [RNA+]) for longer periods and perhaps increase their ability to transmit SNV. In this study, we investigate this question by using recently published RT-PCR assays to screen for SNV RNA in blood samples from antibody-positive deer mice [9]. The samples were collected in Montana over an approximately 1.5-year period from February 2011–June 2012. Our results indicate that SNV Ab+ male *P. maniculatus* are more likely to have circulating RNA than SNV Ab+ females; a finding that might provide an additional explanation for why SNV transmission in the wild appears male dominated.

## 2. Results

Blood samples from known SNV Ab+ mice were analyzed using two RT-PCR assays, each optimized for S and M genomic segments of SNV strains known to circulate in Montana [9]. Of 78 SNV Ab+ samples, representing 56 individual mice, the overall prevalence of samples positive for SNV RNA was 77%, with 74% of these positive for both segments (Table 1). On a per mouse basis, in which repeat samples from the same mouse were excluded from the dataset, similarly high levels were observed (75%). These prevalence data overall are 3–4 times greater than that found in a similar study of Ab+ samples from mice caught in the same region [7] and these data appear consistent across locations (66%–100%; Table 1). A major difference between Kuenzi *et al*., [7] and the current study is that Kuenzi *et al*., [7] used different RNA extraction methods and a RT-PCR assay that was not optimized for local Montana strains (see below).

**Table 1:** Prevalence of detectable viral RNA in Sin Nombre virus antibody-positive blood samples from North American deer mice from southwestern Montana, USA, 2011–2012. *Ab+*: *antibody positive*, *RNA+*: *positive for viral RNA by RT-PCR*.

	Number (%) of total Ab+ samples RNA+ for either segment	Number (%) of total Ab+ samples RNA+ for both segments	Total Ab+
**Ab+ blood samples**	61 (77)	45 (58)	78
**Individual mice^a^**	42 (75)	31 (57)^b^	56^a^
**Males**	30 (86)	25 (71)	35
**Females**	12 (57)	7 (33)	21
**Recaptured mice^c^**	10 (77)	9 (75)	13
**Males**	9 (100)	9 (100)	9
**Females**	1 (25)	1 (25)	4
**Ab+ blood samples by location**			
**Cascade**	20 (80)	15 (60)	25
**Gregson**	12 (100)	10 (83)	12
**Polson**	28 (66)	20 (49)	41

^a^ excludes 2 mice that had varying RNA positivity results. ^b^ excludes 2 mice that had varying results regarding S segment detection on different recapture dates. Denominator was 54 instead of 56. ^c^ mice that had 2 consecutive capture dates.

To determine if the increased levels of detection we found were due to more sensitive methods rather than a temporal spike in virus prevalence during the 2011–2012 collection period, we tested 20 archived SNV antibody-positive samples collected from the same region in 2007 and 2009. These archived samples were originally analyzed using the RNA extraction method [20] and a standard nested RT-PCR assay based on the M segment of a SNV strain from New Mexico, USA (strain NMR11; Genbank Accession# L37904). We found 75% (15/20) of these archived blood samples positive for SNV RNA compared to only 5% (1/20) in the initial testing, indicating the detection methods used here were more sensitive.

The increased ability to detect low levels of RNA allowed us to further investigate the degree to which previously infected mice show evidence of long-term circulation of viral RNA, and whether or not sex plays a role in this process. We first examined Ab+ mice caught for two consecutive months (n = 13), and found the vast majority, 77%, to be RNA+ each time (Table 1). Extending this analysis to mice caught beyond two months was difficult due to the low numbers of Ab+ animals consecutively caught during the 2011–12 period (n = 3). However, by also including samples from consecutively sampled Ab+ mice captured in 2007 and 2009 for which sufficient blood volumes were still available (n = 3), we found 67% (4/6) of mice to be RNA+ every time they were captured (Table 2). One of these was RNA+ on four consecutive capture dates and again when caught seven months later (mouse 5), indicating that he had detectable viral RNA approximately a year past his first capture. Finally, there is evidence that sex plays a role in the ability of *P. maniculatus* to clear SNV virus from the bloodstream. As shown in Table 1, 71% of RNA+ mice were males (30/42), *versus* 29% (12/42) for females. By sex, 86% of SNV Ab+ males have detectable viral RNA in their blood (30/35) *versus* 57% (12/21) for females (Pearson *x*^2^ (1, n = 56) = 4.66, p = 0.03083). Further, in persistently ‘viremic’ animals (e.g., those mice RNA+ for two consecutive months), 90% were males (9/10) *versus* only 10% for females (1/10), and by sex, 100% (9/9) of Ab+ males were viremic *versus* 25% (1/4) for females. For those mice RNA+ for three or more consecutive months, 100% (4/4) were males (Table 2), including the mouse tested and found positive nearly a year from his initial capture.

**Table 2:** Detection of Sin Nombre virus RNA in antibody-positive North American deer mice captured and sampled on three or more monthly trapping events.

Mouse # (Tag)	Sex	RNA + Initial Capture	RNA + Recaptures	Recapture time span (months)
1 (C408)	Female	Y	N	3
2 (D4370)	Male	Y	N	3
3 (H183)	Male	Y	Y	3
4 (D4344)	Male	Y	Y	4
5 (D5433)	Male	Y	Y	4
6 (D6536)	Male	Y	Y	6

## 3. Discussion

Using geographic region-specific RT-PCR assays to screen for viral RNA in blood samples of SNV Ab+ North American deer mice, we found 75% to be RNA+ with some mice showing evidence of sustained circulating RNA for 4–6 months. These data demonstrate a much higher prevalence of circulating RNA than in previous SNV studies in Montana and Canada where 19%–47% of mice were RNA-positive ([7,8], respectively). Our results are most consistent with previous experimental infection studies in which SNV RNA was detected beyond two months in the majority of infected animals [6], perhaps due to the fact that they too used an efficient RT-PCR detection assay optimized for the SNV strain used as the virus inoculum. We used non-degenerate primer sets that were highly conserved among SNV variants from Montana resulting in increased detection of region-specific SNV strains and a modified extraction method that worked well for purifying RNA from low-volume blood samples.

A major result from this study was the discovery that SNV-Ab+ male deer mice are significantly more likely than females to have detectable viral RNA in their blood, 86% *versus* 57%, respectively. This trend became more pronounced when examining mice with persistent RNA circulation; for Ab+ animals captured in consecutive months, males were more than twice as likely as females to be RNA+, 100% *versus* 25%. The biological reasons for this bias are unclear, but may be related to differential regulation of sex-specific hormones like that seen with SEOV and Norway rats. In those studies, infected male rats had higher levels of viral RNA in their lungs than females, were more likely to shed virus through saliva and excreta, and had diminished expression of genes related to the innate antiviral immune response [21,22]. This sex-specific phenotype was reversed upon performing gonadectomies in both sexes, whereupon females shed more virus and males less virus in comparison to their intact counterparts [22]. The immune system of *P. maniculatus*, in response to SNV infection, has been recently probed and may soon be amenable for study of sex-specific immune regulation. Using experimentally infected deer mice, Schountz *et al*., [19] detected neutralizing antibody at very low levels during the first week PI. In addition, T-cells isolated from SNV-infected deer mice included components of immunosuppressive regulatory T-cell activity, which can down-regulate inflammatory responses [23]. The limited presence of neutralizing antibody and apparent down-regulation of the immune system by SNV infection [19,23], result in an immunological environment that allows viral persistence in the blood well beyond the first 21 days of infection. Unfortunately, the authors did not report their results according to sex, thus making it impossible determine any influence of sex on the length of viremia. Our results suggest that, in addition to behavioral differences between sexes, sex‑based distinctions in the progression of SNV infection (through an immunologic/physiologic mechanism) may be an important determinant of virus transmission in deer mouse populations. These two mechanisms together may be collectively responsible for the higher prevalence of SNV infection in male deer mice.

Because we did not know the exact date mice became infected, we could not determine if their infections were within the acute or persistent phase. In addition, although it is likely that infectious virus is circulating in the blood, that has not been demonstrated specifically here (e.g., the viral RNA we detected may be virus bound by antibody). Experiments in at least one other New World hantavirus-host system showed that viral RNA levels detected in blood mimicked patterns of infectious virus in the blood and tissues, including the salivary glands [24]. 

Twenty-three percent of the Ab+ mice were negative for SNV RNA. This may be explained by the fact that 80% of the RNA-negative (RNA-) samples were from large adults (23–27 g), raising the possibility that these individuals were infected in a previous season but not captured and tested, a likely consequence of less frequent monthly recapture studies. Alternatively, it is known that the antibody test for SNV is highly cross-reactive with other North American hantaviruses, and therefore we cannot rule out that some of the Ab+ but RNA- mice were infected with other hantaviruses such as Prospect Hill virus, hosted by meadow voles, *Microtus pennsyvlanicus*. At these sites, deer mice often share habitat and burrows with *Microtus* spp*.*, and inter-species virus spillover has been documented in other deer mouse-SNV systems [25]. Finally, infected mice could be displaying differential patterns of infection as suggested by various experimental infection studies that categorized SNV-infected mice as having either “disseminated” or “restricted” infection patterns within the tissues during either the acute or persistent phases [6,23]. Depending on the type of infection, virus may or may not be present in the blood stream. Of note, donor mice that successfully infect other mice tended to have higher levels of RNA in their blood [9]. 

## 4. Experimental Section

We obtained blood samples from an ongoing 18-year monthly longitudinal mark-recapture study at three sites in Montana (Table 1; see [7]). Blood collection is described by Kuenzi *et al*. [7]. When collecting blood samples, males and females were handled in a random order, thus preventing any contamination bias by sex. Monthly blood samples were tested for SNV antibody as described by Schountz *et al*. [26]. The majority of Ab+ mice were adults (95%); only three mice were subadults. We used a protocol that combines a guanidinium thiocyanate-phenol-chloroform extraction with a Qiagen RNAEasy kit (Qiagen Inc., Valencia, CA, USA) [9] to extract total RNA from all SNV Ab+ blood samples collected February 2011–June 2012. We screened each sample for the small (S) RNA segment which encodes the nucleocapsid protein and medium (M) segment which encodes two glycoproteins. We used the RT-PCR assay from Bagamian *et al*. [9], with some modifications. We used primer sets designed from sequence regions that are conserved across all Montana strains. Our screening RT-PCR for the M segment consisted of sense primer M1772L and antisense primer M2648R [9]. The S segment primers were sense primer: S208L-5'- ACCAAACTCGGAGAACTCAAAC-3' and antisense primer: S1094R-5'-, TCAGATGTTCCCACAGATTTTG-3. The procedure was largely as published [9]. Briefly, we used 5 uL of total RNA extracted from blood samples in RT-PCR assays with the Superscript III One-Step RT-PCR with Hi Fidelity Taq Kit (Invitrogen, Carlsbad, CA, USA). The RT and cycling conditions were: cDNA synthesis: 55 °C for 30 min, pre-denaturation at 94 °C for 2 min, followed by 40 cycles (45 cycles for primer set S208L/S1094R) at 94 °C for 15 s, 55 °C for 30 sec, and 68 °C for 1 min, and a final extension at 68 °C for 5 min. PCR products from 2012 samples were purified and sequenced using the PCR primers or internal sequencing primers by Beckman Coulter Genomics (Boston, MA, USA) to confirm identity (data not shown). We used the program Primer3 to design all RT-PCR primers and Microsoft Excel and R for statistical analyses. We also tested 20 archived SNV Ab+ samples that were previously tested using a non-geographic region specific protocol for comparison (see results). These samples were from the same three sites as the 2011–12 samples, plus one additional site (Butte). There were at least three samples from each location. There were eight different mice tested with their masses ranging from 17–26 g; all were adult males except for one adult female and one subadult male. Four samples were from 7/08–10/09, the rest were from 5/07–11/07.

To prevent cross-contamination, RNA extractions were conducted in a separate class II biosafety cabinet with biosafety level-3 precautions. We handled all PCR amplicons in a separate laboratory with equipment and supplies solely dedicated to their analyses. PCR amplicons were genetically distinct (data not shown), and most closely matched those from mice sampled previously from the same region.

## 5. Conclusions

We found evidence that males are more likely to be circulating RNA in their blood as compared to females. Future research should explore the relationship of the immunological, physiological, and ecological factors that can influence the pattern of infection, including the role of testosterone and estrogen and their relationship to hantavirus transmission-related behaviors and host immunology and pathology in deer mice. Also, further study should focus on exploring viral RNA and antibody levels, as well as other related immune system components, in response to natural SNV infection in males and females to understand the relevant roles of each sex in the maintenance of SNV transmission in the wild. 
